# Conflict Sources and Management in the ICU Setting before and during COVID-19: A Scoping Review of the Literature

**DOI:** 10.3390/ijerph19031875

**Published:** 2022-02-08

**Authors:** Katarzyna Czyż-Szypenbejl, Wioletta Mędrzycka-Dąbrowska, Anna Falcó-Pegueroles, Sandra Lange

**Affiliations:** 1 Department of Anaesthesiology Nursing & Intensive Care, Faculty of Health Sciences, Medical University of Gdansk, 80-211 Gdansk, Poland; katarzyna.czyz-szypenbejl@gumed.edu.pl; 2 Department of Fundamental Care and Medical-Surgital Nursing, School of Nursing, Faculty of Medicine and Health Sciences, University of Barcelona, 08036 Barcelona, Spain; annafalco@ub.edu; 3 Department of Internal and Pediatric Nursing, Faculty of Health Sciences, Medical University of Gdansk, 80-211 Gdansk, Poland; langa94@gumed.edu.pl

**Keywords:** ICU conflict, ICU environment, practical management, multi-professional team

## Abstract

Introduction. Conflicts are an inherent part of work within any organisation. They can arise between members of an interdisciplinary team (or between teams representing different departments), between patients and team members/family members, and patients’ families and team members. Various conflict situations among employees may occur, therefore it is very important to identify their causes and take preventive or targeted corrective measures. The aim of this study was to review the available literature concerning conflicts arising in ICUs—their types, methods of expression as well as their management and mitigation. In addition, we reviewed the available literature on the impact of the pandemic on the ICU environment caring for COVID-19 patients. Methods. The databases were searched. Single key words or their combinations using AND or OR operators were entered. Eventually, 15 articles were included in our review, which included two identical papers. Results. Conflicts occurred occasionally or rarely; researchers describing ethical conflicts demonstrated a moderate level of exposure to conflicts. The pandemic created many challenges and ethical dilemmas that are a source of ethical conflict. Conclusions. As conflict by nature remains inevitable, adequate procedures in conflict management should be developed and the leadership of managing personnel should be reinforced, because team members frequently expect guidance from their supervisors. The importance of training in interpersonal communication and crisis situation management in healthcare should therefore be emphasised.

## 1. Introduction

Conflicts are an inherent part of work within any organisation and may be defined as a situation in which opposing people, who support different solutions, participate [1,2]. They can arise between members of an interdisciplinary team (or between teams representing different departments), between patients and team members/family members, and patients’ families and team members [1]. They may concern the decisions that families surrogates [3] have to make or stem from organisational problems or from the individual characteristics of team members [4]. The intensive care unit (ICU) is a very specific workplace, where the most severely ill patients are admitted, all in life-threatening conditions. The multitude of diseases/injuries and medical equipment, the amount of work, time pressure as well as physical and mental strain may lead to controversy between the members of the therapeutic team and other personnel, occupational burnout [5,6,7] and may be a cause of ethical conflicts [8]. The frequency and intensity of ethical conflicts increased during the current pandemic for COVID-19. Isolation, the large number of critically ill patients raised ethical concerns not only the withdraw life support decision but also regarding the involvement of the family in caring for and accompanying the patient at the end-of-life [9]. In addition, the highly infectious nature of the COVID-19 virus causes concern for one’s own health, which contributes to the conflict between the interests of the patient and the safety of medical personnel and their families. Particularly with the limited availability of material resources such as PPE (Personal Protective Equipment) [10]. These factors can shake up our ethical principles and sharpen our ethical dilemmas [11]. Unfortunately, these circumstances have increased conflict situations from an ethical perspective in already complex situations in some health systems. To deal with these challenging circumstances, it requires in-depth knowledge of the subject and management abilities as well as capacity to prevent conflict, whether or not there is an unusual situation such as a pandemic.

Conflict is commonly perceived as a negative phenomenon because if it is poorly managed, it may result in low job satisfaction, and staff turnover. On the other hand, once the conflict is quickly identified and managed, it may have a positive outcome [3]. Therefore, effective conflict management is crucial for the functioning of an ICU, as collaboration and shared decision-making are basic features for success in this field of medicine [12], nursing and health care.

There are many definitions and types of conflict described in the literature, which concomitantly identifies five basic methods of conflict management [13]: competition, collaboration, compromise, avoidance, and accommodation. This classification is based on two independent poles of motivation: the maximisation of one’s own benefit or the maximisation of a partner’s benefit. It should be emphasised that none of these styles has any advantage over the other, and the approach a given person presents depends on her/his own preferences, which could originate from experience gathered during previous conflicts [13].

Personality, i.e., the combination of psychological features of a given person, may also be one of the behavioural determinants during a conflict [14]. This may be of great importance because due to the high qualifications required to work in an ICU, the employees are usually mentally tough. Escalation is one of the ways a conflict may develop, which is most commonly associated with a non-conscious reason for a disagreement. When emotions prevail over relations, it leads to a situation when the essence of the matter vanishes, and the conflict becomes personal. Another direction of management, i.e., the search for a solution, develops when parties concentrate on the reasons for the conflict. Reconsidering the essence of the problem may lead to concessions and a transformation of the need to compete into collaboration or accommodation. On the other hand, when the relation breaks, it is a result of the lack of possibility to reconcile one’s own values with the values presented by another person, which in turn may lead to the creation of a “mask” of correct relations. The first condition for effective conflict management is the realisation of impending litigation, in order to take appropriate preventive actions. Warning signals may include: disturbances in communication, stereotypes or prejudices, different values, or potential interests of one of the parties. The second condition for successfully dealing with conflict is a thorough analysis of the situation, taking into account the characteristics of its active participants, and the scope, causes and intensity of the conflict—the interpretation of which will enable the selection of appropriate measures and methods to solve existing problems or even strengthen the positive effects by bringing participants closer to each other [15]. Emotionally stressful situations require strong emotional intelligence from managers [16]. As many conflicts may arise among ICU staff, it is very important to identify their causes, to take preventive measures adequately early or to introduce targeted corrective measures. Even more so when there is a situations of high demand for the resolution capacity of professionals, managers and health systems, such as the current COVID-19 pandemic.

In this line, the aim of this study was to review the available literature concerning on conflict sources and management in the ICU setting before and during COVID-19 pandemic.

## 2. Method

### 2.1. Study Design

A review was carried out from April 2020 to November 2021.

### 2.2. Search Methods

The following databases were searched: EBSCO, Ovid, PubMed, Web of Science, ProQuest, Cochrane. The following key words were used: “conflict”, “intensive care unit (ICU)”, “nurses”, “physicians”, “conflict management”. Single key words or their combinations using AND or OR operators were entered. A total of 4001 papers were found and 42 were selected for further analysis, which included verifying the availability of the full-text versions, and their match with the inclusion criteria. The last search was conducted on 25 November 2020. Eventually, 8 articles were included in our review. During the time we were working on the review, the COVID-19 pandemic arrived, which significantly affected changes in the ICU environment and complex ethical issues. Therefore, our search comes from two different periods, i.e., first search ranged between years 2015 and 2020 and refers to conflicts in the ICUs not related to the pandemic. The second search concerned the period after COVID-19 pandemic occurred (2019–2021). The search, related with COVID-19 pandemic period, was performed via PubMed. The following key words with AND or OR operators were used: “conflicts”, “nurses”, “physicians”, “intensive care unit (ICU)”. The last search was performed on 5 November 2021. A total of 411 papers were found and after verification following inclusions and exclusion criteria, 7 articles were included in review. Finally, 8 articles from pre-COVID-19 era and 7 articles since the pandemic outbroke were included to the review. The search strategy was determined by ethical nature of conflicts, which escalated during the COVID-19 pandemic.

### 2.3. Study Selection

Inclusion criteria:

- Year of publication: 2015–2021 (the researchers focused on the latest studies, hence the years of publication);
- Studies carried out in intensive care units for adults;
- Publication type (original papers only).

Exclusion criteria:

- Year of publication earlier than 2015;
- Studies carried out in departments other than ICUs;
- Articles concerning neonatal and paediatric intensive care units;
- Publication type (articles with research examples, letters to the editor, meta-analyses, review papers).

### 2.4. Research Variables and Strategy

Descriptive data is presented in the form of a table presenting: author and year of publication, participants, research group, research instrument, conflict type and results (Table 1).

### 2.5. Methodological Quality and Level of Evidence

All papers selected for inclusion in the systematic review were subjected to appraisal by two independent experts in the field of critical care. The Joanna Briggs Institute JBI Critical (Supplement 1) appraisal checklist were used to assess the methodological quality of the study and study possibility of bias in its design, conduct and analysis [17]. The assessment process are presented in Table 2.

### 2.6. Ethical Aspects

The consent of the bioethical commission was not needed to conduct a literature review due to the type of article.

## 3. Results

A total of 15 papers that met the inclusion/exclusion criteria were selected for final analysis. From 2015–2020 period, 8 met the inclusion criteria; 3 of them described ethical conflicts perceived by nurses. 3 groups of researchers used the Ethical Conflict in Nursing Questionnaire—Critical Care Version [18,19,20], while the other 2 conducted interviews [21,22], 1 used the original questionnaire [23], 1 used the technique of observation and in-depth interview [24], and 1 open-ended questions [25].

Healthcare workers who participated in the researched studies included nurses [18,19,20,25] and physicians [21,22,23]. Other investigators studied patients’ families [24] and auxiliary personnel (social workers, chaplains, case managers) [21].

With regard to conflicts, these occurred occasionally or rarely. Researchers describing ethical conflicts indicated a moderate level of exposure to conflicts. The results are presented in Table 3.

**Figure 1:**
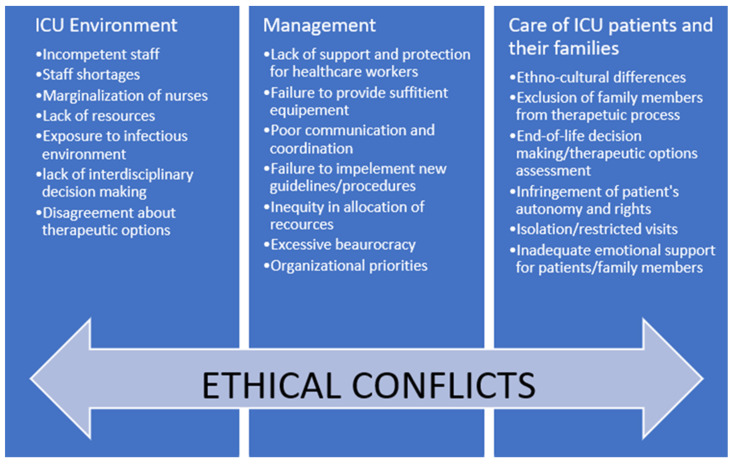
Summary of sources of conflict in the ICUs.

We hypothesized that SARS-CoV-2 pandemic had an impact on the issue of conflicts. From the second, additional search after the outbreak of pandemic COVID-19, 7 articles were selected for final analysis. Study groups included nurses [26,27,28,29], nurses, physicians, RTs (Respiratory Therapists), APPs (Advanced Practice Providers) [10] and nurses, intensivists, supporting staff [30] and five experts from working group [31]. Most researchers used a semi-structured interview technique [26,27,28,29]. One research group used Measurement of Moral Distress for Healthcare Professionals (MMD-HP) and Ethical Decision-Making Climate Questionnaire (EDMCQ) [30], one used a nominal group technique [31] and one structured questionnaire [10]. In contrast to the pre-COVID-19 study, during the pandemic all participants struggled with conflicts caused by ethical challenges and dilemmas. A summary of main findings regarding sources of conflict before and during the COVID-19 pandemic that, if unrecognized and unresolved, may cause ethical conflict in the ICU are presented in Figure 1. We categorized these sources into three areas related to: (1) ICU Environment, (2) Care of COVID-19 patients and their families, and (3) Management.

## 4. Discussion

In nursing, conflicts most commonly concern organisational factors, interpersonal disagreements, or individual features (immature personality) [32]. The epidemiology of conflicts in an ICU is well described. Schuster et al. study showed that conflict between physicians and surrogates is common in intensive care units (63% of cases) [33]. The study carried out by Paprocka-Lipińska et al. found that conflicts in ICU occur rarely (25%) or occasionally (43%) [23], which was confirmed by other authors [18,19]. As the literature shows, the pandemic has caused many changes in the care of ICU patients. This has resulted in increased conflicts and ethical dilemmas. Therefore, it is important to understand the sources of conflict for introducing improvements that will reduce negative effects among medical professionals, healthcare institutions, and most importantly, the quality of care for critically ill patients.

**Figure 2:**
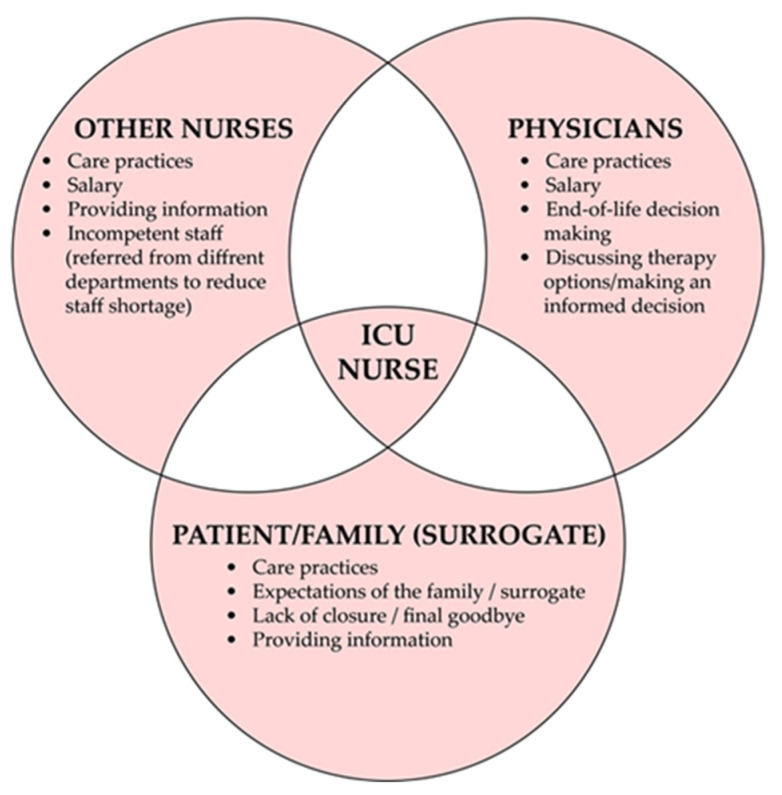
Common sources of conflicts in ICUs.

Conflicts within an ICU involve the process of taking care of a patient, emotional commitment, providing information or making decisions concerning futile medical care [23]. Differences in opinions, experience or expectations frequently lead to moral distress [18,21,22,23,24,25]. Nurses and physicians may not share the same view on the aims of the therapy, and the former try to advocate in the best interests of their patients, take holistic care over them and can more easily perceive extra-medical aspects which relate to the well-being of their patients [19,20,34]. Participants included in the study carried out by McAndrew et al. articulated that therapy which is not beneficial for the patient causes suffering and ethical conflicts, Figure 2 [25].

The latter issue is analysed particularly in nurses [19,20,21,22,25]. Ethical conflicts may occur when there is a conflict concerning moral principles, values and beliefs between nurses and the patients or their families [35]. The intensity of this type of conflict increased during the current pandemic, when the great inflow of patients raised ethical concerns not only surrounding triage and the withdrawal of life support decisions, but also regarding family visits and the quality of end-of-life support. These components are liable to shake up our ethical principles, sharpen our ethical problems and lead to situations of major caregiver suffering [11].

Before the pandemic, conflicts between medical professionals and their families were common. These were mainly due to different family expectations and perceptions of ‘good’ care and support for the patient [24].

During the pandemic, visitation policies and the care provided to patients and their families changed [28]. The use of personal protective equipment (PPE) and isolation made it impossible to provide holistic nursing care [26]. Additionally, the isolation necessity made it difficult to use translation tools with patients and their families who did not speak English [28].

The lack of a chance to visit, to accompany in the last moments, and to say goodbye to a family member led to the evaluation of mourning into a pathological type, as highlighted by the nurses in the study by Fernández-Castillo et al. In addition, the nurses pointed out that mourning during the pandemic was experienced not only by family members, but also all staff [26]. According to the Donkers et al., study, the factor “Inadequate emotional support for patients and their families” was regarded as most morally distressing by all professions [30].

Already before the outbreak of the pandemic, lack of adequate equipment and material resources was causing conflicts. A study conducted in South Africa by Ramathuba et al. showed that healthcare professionals were not able to provide high-quality care due to limited resources, including the availability of hospital beds and some medical equipment, which in turn led to ethical dilemmas [22]. In a global study by Wahlster et al., a shortage of beds in COVID-19 ICUs was reported by 13% of respondents, with most shortages (50%) alarmed in South Asia [10]. Limited access to ventilators was reported by 7%-43% of respondents [10]. In a study by Jia Y. and Falcó-Pegueroles A. et al. observed that the limitations in medical resources and the restrictions imposed resulted in the neglect of some of the patient’s rights. For example, the right to choose a treatment plan, the right to information or the provision of end-of-life care. One interviewee confessed that sometimes in order to prevent anxiety and worsening of the patient’s condition, he hid the truth from them, which contributed to his ethical conflict [27,31]. The nurse from the study by Liu et al., mentioned that she did not allow the patient to see his blood oxygen saturation index so as not to exacerbate his stress [29]. Furthermore, the highly infectious nature of the COVID-19 virus contributed to the ethical conflict between the interests of the patient and the nurse’s own safety, as exposed in study by Jia Y. et al., Liu X. et al., and Falcó-Pegueroles A. et al. [27,29,31].

The lack of qualified personnel raised moral distress, as well [22]. Both pre-pandemic studies by Saberi et al. [20] indicate that the situation that caused the most conflict was associated with working with incompetent staff, which is supported by the findings of Pishgooie et al. and studies conducted during the pandemic by Donkers et al. [19,30].

After the outbreak of the pandemic, many hospitals struggled with a shortage of intensivists and ICU nurses [10]. This made it necessary to deploy medical teams from different hospitals to areas with the greatest need for specialists. The unfamiliarity of the new environment, co-workers, and differences in terms of work habits may have contributed to interpersonal conflicts. However, a study by Liu X. et al. reported that ICU teams, where work was based on a seamless collaboration gained approval from other staff as well as increased work efficiency [29]. Similarly, in the study by Fernández-Castillo et al., and Gordon et al., ICU staff reported strengthening collaboration, creating an atmosphere of safety, solidarity, and support for each other while working in the ICU for COVID-19 patients [26,28]. In the Donkers et al. study, factor analysis showed that ethical climate factors such as: “Culture of mutual respect within the interdisciplinary team” and “Practice and culture of ethical awareness and support” were most positively among the staff [30]. The pandemic also revealed inequality in treatment, as well as role ambiguity between nurses and doctors. Studies showed that nurses spent significantly more time in the infectious environment. In addition, some of the doctors expected nurses to take over some of the duties e.g., lung auscultation. This caused nurses to feel inferior and also disrespected [27]. Similarly, 22% of respondents from the Donkers et al. study, highlighted the top-down nature of the relationship and the presence of hierarchy, and 17% the feeling undervalued [30]. Nurses in the Fernández-Castillo study also mentioned overlapping professional roles and responsibilities. They performed tasks that did not correspond to their qualification e.g., modification of parameters in the respiratory or during the procedure, the nurse put in an arterial line and the doctor gave her materials. However, it is important to highlight that mutual collaboration, had a positive impact on the experience in the aspect of teamwork during the COVID-19 pandemic among Spanish ICU nurses [26]. The pandemic forced the delegation of staff from other hospital departments, with no previous experience of critically ill patients, to work in the ICU. This compounded ethical conflicts about the quality of care and dilemmas in the relationship nurse-nurse, as showed the Fernández-Castillo et al. study [26].

McAndrew et al., in a study before the pandemic, identified three groups of conflicts, i.e., the practices and organisation of ICUs which have an impact on the perception of the patient’s suffering; the marginalisation of nurses during conflicts; the engagement of hospital resources to reduce moral distress (communication and collaboration with other staff members who could act as an ethical guide, i.e., social workers) [25]. Falcó-Pegueroles et al. indicated that the most morally pressing issues include the lack of engagement in making decisions about the patient, and inefficient therapy, which was also described in later studies [20,21,27]. The results of these studies carried out by both Saberi et al. and Falcó-Pegueroles et al. demonstrated that the continuation of therapy upon the family’s request and against the patient’s will was the least significant factor behind ethical conflicts in ICU nurses [18,20]. On the other hand, Van Keer et al. emphasised that patients’ families (being in opposition to ICU personnel) perceived the extra-medical needs of the family members more often, which resulted in conflict between these two groups. Van Keer et al. hypothesised that ethnic and cultural differences could be responsible for ICU conflicts, yet the conflicts appeared to occur due to universal problems, e.g., the structure/statute/customs of the given ICU [24].

**Figure 3:**
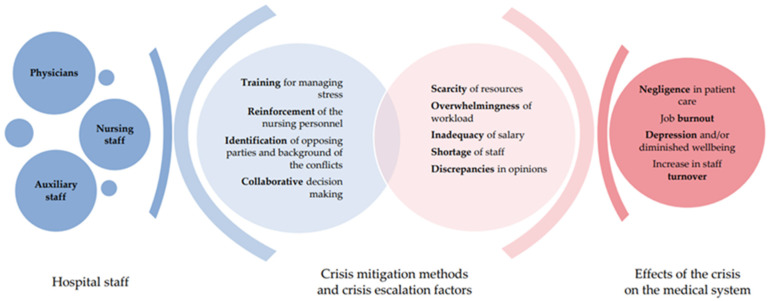
Cause and effect relationship in conflict management.

Isolation, limited resources and staff shortages, large number of patients have resulted changes in previous guidelines for decision-making regarding ICU admissions, CPR (Cardiopulmonary Resuscitation), eligibility for respiratory therapy, end-of-life care, decisions regarding discontinuation of therapy and family involvement in decision-making [10,31]. Changes in practices due to the pandemic were reported by 66% of respondents from a global survey by Wahlster et al. Factors such as clinical severity, co-morbidities, and patient age were considered by CPR decision-making. The majority of respondents to this survey reported that the physician made the CPR decision. Only in the North American region, 67% of CPR decisions were made at the request of the patient’s family. Family involvement in decision-making about withdrawing or withholding life-sustaining therapy has also decreased [10]. 48% of respondents from the Donkers et al. study, indicated a lack of interdisciplinary decision making and 8% a lack of nurse engagement in end-of-life care decisions for patients with COVID-19 [30]. The omission to comprehensively analyse the available treatment options, the lack of engagement of all persons and respect for the wishes and rights of the patient generate ethical conflict in the ICU for COVID-19 patients [31]. The pandemic caused changes in end-of-life care for critically ill patients. Due to strict isolation restrictions, accompanying the patient in the terminal period of life, relieving pain and suffering has been limited. In addition, accompanying the family through the dying process, goodbyes were prevented by the restrictions enforced by the pandemic, Figure 3 [31].

Falcó-Pegueroles et al. demonstrated that there is a connection between frequent exposure to ethical conflicts in stressful environment and consideration of turnover [19], which is consistent with the results obtained by Embiaco et al., who pointed out that work pressure and conflicts with co-workers resulted in occupational burnout, which is a factor driving turnover [5]. This was also mentioned in the most recent study by Shaffer et al., who demonstrated that poor relationships between nurses and physicians may be a factor responsible for job changes [36]. On the other hand, the study by Paprocka-Lipińska et al. revealed that financial issues, most commonly inadequate salaries, are the primary factor contributing to conflicts between nurses and physicians. Other factors which commonly lead to conflicts include work pressure and excessive bureaucracy [23]. Economic factors were also found to be a source of ethical conflicts in the study carried out by Saberi et al., who noticed that bonuses were not merit-based, which could have an impact on motivation and cause a feeling of worthlessness [20]. Interestingly, Paprocka-Lipińska et al., observed that non-compliance with ethical rules was rarely a reason for conflicts (almost 74% of respondents gave such an answer). Conflicts with patients’ families turned out not to be common as well, which may result from the specific nature of Polish ICUs, as patients’ family members are usually not asked for their opinions, and physicians take all the responsibility for the continuation or discontinuation of the therapy [23]. Interestingly, during the pandemic, the economic factor fell by the wayside. A significant minority reported concern about their financial situation-11% [10]. However, new factors arose that raised ethical challenges. On the one hand, healthcare workers were seen as heroes, on the other hand, it was stigmatizing (people feared for their own safety while meeting HCWs). Many interviewees highlighted the fact that categorising HCWs (Health Care Workers) as heroes caused internal conflict, as they did not share this feeling [28]. In the Wahlster et al. study, 21% reported fears of being stigmatised by society, which is compliant with the Gordon et al. study, in which workers also had to deal with social challenges [10,28]. McAndrew et al. noted that nurses asked their supervisors for help in resolving conflicts, yet did not receive such help in every case (managing personnel had no time to resolve conflicts) [25]. As regards dealing with a problem, Bruce et al. presented several methods for resolving a conflict—maladaptive (pas-de-deux, fighting, desensitisation) and constructive behaviours (venting, mentoring, building team cohesion). Some people revealed that gallows humour helps them reduce tension during emotionally stressful situations—the use of satire enabled them to protect themselves from the effects of discordance [21]. In the light of the pandemic, the role of supervisors appears to be particularly important in resolving ethical dilemmas and conflicts. A large number of respondents from the Donkers et al. study, declared that professional support was provided to them in case of moral distress and indicated the openness of managers. However, comments reported by ICU nurses included poor communication about rules, regulations, and guidelines from supervisors, which was a source of moral distress [30]. Poor communication from supervisors, increased the probability of burnout by 30% in a study by Wahlster et al. [10]. The methods of managing conflict were not studied by other researchers. It should be noted that a further search for solutions for conflict management is required. The expectations of personnel regarding conflict management involve supervisors taking control, who are not, however, able to perceive every conflict. Furthermore, inadequate relations between physicians and nurses may contribute to open hostility. Intervention by a supervisor plays a key role in such situations, because disrespectful behaviour has a negative impact on every team member, Figure 3 [37].

Identifying the ethical conflicts that occurred during the COVID-19 pandemic and determining their source should become a priority for researchers to implement remedies as soon as possible, as well as to develop strategies for the future. This will help not only to better prepare for crisis situations but also to minimise the negative effects caused by ethical dilemmas and conflicts among front-line staff. A survey among intensivists of The European Society of Intensive Care Medicine (data from 85 countries) showed that the prevalence of symptoms of anxiety, depression, severe burnout was 46.5%, 30.2% and 51% respectively [38]. Similarly, in a study among ICU staff caring for COVID-19 patients, symptoms of anxiety, depression, post-traumatic stress disorder and burnout were present in 60.0%, 36.1%, 28.4% and 45.1% of respondents, respectively [39]. The Italian Ministry of Health reports that more than 18 thousand health care workers have been infected with SARS-CoV-2, 150 have died. In Spain, more than 16 thousand workers have been infected and 30 have died. In addition, two nurses committed suicide due to excessive stress related to working conditions [31]. Future research will show how severe mental health disorders the pandemic will cause among HCW workers fighting on the front line. Finally, the question arises: will mental illness and burnout caused by the pandemic be a factor compounding medical staff shortages, particularly in ICUs?

## 5. Limitations

The studies included in the review come from different regions of the world. Thus, variables must be taken into account: economic, social, cultural, technological, which may influence both the reasons why conflicts occur and how they are resolved. For example, in South Africa, even before the pandemic, health workers faced challenges of resource scarcity and poor decision-making in resource-poor settings, which caused ethical dilemmas. Following the COVID-19 pandemic, most of the country faced the challenge of providing care to patients despite limited resources, which also led to ethical dilemmas. However, the proportion of respondents reporting limited resource availability still varied by region, as shown in the study by Wahlster et al. Similarly, ethical conflicts stemmed from end-of-life decisions made during the pandemic. These differ according to the cultural conditions prevailing in the region before the pandemic (decisions based on the wishes of the family versus only the doctor’s decision [10].

The results therefore cannot be considered in general terms, the existence of variables that differ from region to region must be taken into account.

## 6. Implications for Practice

Conflicts are an inherent part of work within any group, therefore actions should be taken to minimise the risk of occupational burnout, staff turnover, and to increase the morale of the entire team. The correct identification and management of conflict may have positive consequences. We suggest that the following points should be considered while resolving a conflict:

- identification of opposing parties;
- identification of the background of the conflict;
- provide a time and place for the analysis and debate of conflictive situations;
- personnel training on interpersonal communication;
- training on managing stress and relieving emotions, e.g., debriefing;
- training for managing personnel in conflict management;
- reinforcement of nursing personnel in reaching a consensus on the suggested therapy;
- more accessible psychological support;
- clear vision and feedback from management;
- shared decision making;
- improving the implementation of new guidelines and orders.

## 7. Conclusions

There is a variety of studies describing the types of conflict which occur in ICUs, but too few of them address the issue of conflict management. As intensive care environment might be hazardous and oppressive, conflict management is crucial for improvement of working conditions. Many authors approach the ethical background of conflict between physicians and nurses, and intrinsic conflict. This may result from differences in opinions and experience gained during work, and the impossibility to fully satisfy the needs of patients and/or their families. Nurses present a deep sense of responsibility in terms of the well-being of their patients, not only in the physiological, but also the psychological, social, and spiritual areas. This nurse’s responsibility has become much more highlighted during the pandemic. They perceive a patient as a whole and are able to distinguish the boundary between therapy and futile medical care. Their role in the interdisciplinary team should therefore be reinforced. The COVID-19 pandemic has created new challenges and ethical dilemmas in the ICUs. To support HCWs through the hard times and difficult working conditions in ICUs for COVID-19 patients, identifying the factors that give rise to ethical conflicts should become a priority for management. As conflict by nature remains inevitable, adequate procedures in conflict management should be developed and the leadership of managing personnel should be strengthen, because team members frequently expect guidance from their supervisors. Future studies should delve into the importance of training in interpersonal communication, as well as strategies for conflict prevention and management in critical situations, such as current pandemic.

## Figures and Tables

**Table 1:** Search strategy.

Data Base	Search Strings	Search Period	Obtained Articles	Articles Meeting Inclusion Criteria
EBSCO	(conflicts) AND (nurses OR physicians) AND (intensive care unit OR ICU) AND (conflict management)	2015–2020	225	4
Ovid		2015–2020	42	2
PubMed		2015–2020	137	0
Web of Science		2015–2020	16	2
ProQuest		2015–2020	3172	0
Cochrane		2015–2020	3	0
PubMed	(conflicts OR ethical conflicts OR ethical dilemmas OR ethical challenges) AND (nurses OR physicians OR medical staff) AND (intensive care unit OR ICU) AND (pandemic OR COVID-19) AND (conflict management)	2020–2021	411	7
Total		2015–2021	4001	15

**Table 2:** Quality assessment using the Joanna Briggs Institute (JBI) Critical Appraisal Checklist for Cross Sectional Studies/Qualitative Research.

Author/Date	Participants	Research Instrument	Conflict Type	Quality Assessment JBI
Wahlster, S. et al., 2021 [10]	2700 respondents (physicians, nurses, RTs, APPs)	Structured questionnaire	Ethical conflicts	Include
Falcó-Pegueroles, A. et al., 2015 [18]	203 nurses	Ethical Conflict in Nursing Questionnaire–Critical Care Version	Ethical conflicts	Include
Pishgooie, A.H. et al., 2018 [19]	382 ICU nurses	Ethical Conflict in Nursing Questionnaire–Critical Care Version	Ethical conflicts	Include
Saberi, Z. et al., 2018 [20]	216 critical care nurses	Ethical Conflict in Nursing Questionnaire–Critical Care Version	Ethical conflicts	Include
McAndrew, N.S. et al., 2020 [21]	111 ICU nurses	Open-ended questions	Ethical conflicts	Include
Bruce, C.R. et al., 2015 [22]	29 participants (ICU and auxiliary staff)	Interview	Ethical conflicts	Include
Ramathuba, D.U. et al., 2020 [23]	17 healthcare professionals	Unstructured interview	Ethical conflicts	Include
Paprocka-Lipińska, A. et al., 2019 [24]	232 (nurses and physicians)	Original questionnaire	Various conflicts	Include
Van Keer, R.L. et al., 2015 [25]	ICU staff (92) and 10 patients’ relatives	Ethnographic study (descriptive)	Various conflicts	Include
Fernández-Castillo, R.J. et al., 2021 [26]	17 ICU nurses	Semi-structured interviews	Ethical conflicts	Include
Jia, Y. et al., 2021 [27]	18 nurses caring for COVID-19 patients	Interview	Ethical conflicts	Include
Gordon, J.M. et al., 2021 [28]	11 nurses from one ICU	Semi-structured interviews	Ethical conflicts	Include
Liu, X. et al., 2021 [29]	10 nurses, post-deployment to Wuhan	Semi-structured interviews	Ethical conflicts	Include
Donkers, M.A. et al.,2021 [30]	488 ICU Staff (nurses, intensivists, supporting staff)	Measurement of Moral Distress for Healthcare Professionals (MMD-HP) and Ethical Decision-Making Climate Questionnaire (EDMCQ)	Ethical conflicts	Include
Falcó-Pegueroles, A. et al., 2021 [31]	Working group (5 experts)	A nominal group technique	Ethical conflicts	Include

**Table 3:** Descriptive analysis of articles included in the systematic review.

Author/Date	Country	Results
Wahlster, S. et al., 2021 [10]	United Sates of America	Emotional distress or burnout was high across regions and associated with ▪ a shortage of ICU nurses, ▪ reporting a shortage of powered air-purifying respirators, experiencing poor communication from supervisors.
Falcó-Pegueroles, A. et al., 2015 [18]	Spain	Moral distress was caused by: ▪ the limitations of medical resources, ▪ the neglect of the patients’ rights, ▪ the lack of engagement in decision-making process, ▪ inefficient analgesic therapy.
Pishgooie, A.H. et al., 2018 [19]	Spain	Ethical conflicts occurred mostly when: ▪ working with incompetent staff, ▪ continuing futile care ▪ lacking the equipment/time discouraging nurses to give timely information to the patient/family
Saberi, Z. et al., 2018 [20]	Iran	Ethical conflicts mostly occurred when working with incompetent physicians/nurses/nurses assistants; high exposure to ethical conflict appeared within poor organizational culture and management.
McAndrew, N.S. et al., 2020 [21]	United States of America	Ethical dilemmas were a result of: ▪ a lack of limits of medical care (inappropriate/futile care) ▪ a belief that nurses had no control over the patient’s care (nurses were overlooked by physicians) a lack of resources
Bruce, C.R. et al., 2015 [22]	United States of America	Moral distress occurred in situations: ▪ regarding discontinuation of treatment, ▪ the lack of disclosure about interventions.
Paprocka-Lipińska, A. et al., 2019 [24]	Poland	Most common conflicts concerned: ▪ inadequate salary ▪ job overload ▪ excessive bureaucracy
Ramathuba, D.U. et al., 2020 [23]	Republic of South Africa	Ethical conflicts occurred when: ▪ healthcare professionals were unable to provide the best care ▪ administrative constraints caused incapacity to help patients there was a shortage of skilled staff
Van Keer, R.L. et al., 2015 [25]	Belgium	Conflicts involved: ▪ care practices, ▪ emotional involvement, ▪ information exchange, ▪ end-of-life decision making.
Fernández-Castillo, R.J. et al., 2021 [26]	Spain	Nursing care has been influenced by fear and isolation, making it hard to maintain the humanization of the health care.
Jia, Y. et al., 2021 [27]	China	Major ethical challenges, conflicts, and dilemma appeared: neglected patient rights, the lack of emotional support, unequal exposure to the infectious environment, role ambiguity between doctors and nurses, insufficient response to urgency requirements of the situation, low sense of responsibility in nursing services, lack of knowledge and skills, inability in psychological adjustment and stress resistance
Gordon, J.M. et al., 2021 [28]	United States of America	Ethical conflicts occurred due to: ▪ the inability to provide human comforting connection, ▪ experiencing patient deaths, ▪ isolation, ▪ PPE concerns (supply, quality), ▪ care delays, ▪ changing clinical practice guidelines, language barriers.
Liu, X. et al., 2021 [29]	China	Three main categories of ethical dilemmas have been identified: ethical dilemmas in clinical nursing, ethical dilemmas in interpersonal relationships, and ethical dilemmas in nursing management.
Donkers, M.A. et al.,2021 [30]	Holland	Inadequate emotional support for patients and their families was the highest-ranked cause of moral distress for all groups of professionals. Moral distress scores during COVID-19 were significantly lower for ICU nurses and intensivists compared to one year prior
Falcó-Pegueroles, A. et al., 2021 [31]	Spain	Factors of ethical conflicts were identified: ▪ availability and management of resources, ▪ protection of healthcare workers, ▪ circumstances surrounding decisions making, end-of-life care, ▪ communication

## Data Availability

The authors declare that the data of this research are available from the correspondence author on request.

## References

[1] Wujtewicz M., Wujtewicz M.A., Owczuk R. (2015). Konflikty na oddziale anestezjologii i intensywnej terapii. Anaesthesiol. Intensiv. Ther..

[2] Weingart L.R., Behfar K.J., Bendersky C., Todorova G., Jehn K.A. (2015). The Directness and Oppositional Intensity of Conflict Expression. Acad. Manag. Rev..

[3] Chiarchiaro J., White D.B., Ernecoff N.C., Buddadhumaruk P., Schuster R.A., Arnold R.M. (2016). Conflict Management Strategies in the ICU Differ Between Palliative Care Specialists and Intensivists. Crit. Care Med..

[4] Forbat L., Mnatzaganian G., Barclay S. (2019). The Healthcare Conflict Scale: Development, validation and reliability testing of a tool for use across clinical settings. J. Interprof. Care.

[5] Embriaco N., Azoulay E., Barrau K., Kentish N., Pochard F., Loundou A., Papazian L. (2007). High Level of Burnout in Intensivists. Am. J. Respir. Crit. Care Med..

[6] Seaman J.B., Cohen T.R., White U.B. (2018). Reducing the Stress on Clinicians Working in the ICU. JAMA J. Am. Med. Assoc..

[7] Vahedian-Azimi A., Hajiesmaeili M., Kangasniemi M., Fornés-Vives J., Hunsucker R.L., Rahimibashar F., Pourhoseingholi M.A., Farrokhvar L., Miller A. (2017). Effects of Stress on Critical Care Nurses: A National Cross-Sectional Study. J. Intensiv. Care Med..

[8] Moon J.Y., Kim J.-O. (2015). Ethics in the Intensive Care Unit. Tuberc. Respir. Dis..

[9] Pattison N. (2020). End-of-life decisions and care in the midst of a global coronavirus (COVID-19) pandemic. Intensiv. Crit. Care Nurs..

[10] Wahlster S., Sharma M., Lewis A.K., Patel P.V., Hartog C.S., Jannotta G., Blissitt P., Kross E.K., Kassebaum N.J., Greer D.M. (2021). The Coronavirus Disease 2019 Pandemic’s Effect on Critical Care Resources and Health-Care Providers. Chest.

[11] Robert R., Kentish-Barnes N., Boyer A., Laurent A., Azoulay E., Reignier J. (2020). Ethical dilemmas due to the COVID-19 pandemic. Ann. Intensiv. Care.

[12] Rose L. (2011). Interprofessional collaboration in the ICU: How to define?. Nurs. Crit. Care.

[13] Kłusek B. Five Styles of Conflict Resolution: A Questionnaire. Czas Psychol. http://www.czasopismopsychologiczne.pl/files/articles/2009-15-kwestionariusz-stylw-rozwizywania-konfliktw.pdf.

[14] Klusek-Wojciszke B. Personality Antecedents of Conflict Resolution Styles (Osobowosc jako Determinanta Wyboru Stylu Rozwiazywania Konfliktów). http://bazekon.icm.edu.pl/bazekon/element/bwmeta1.element.ekon-element-000164805565.

[15] Saryusz-Wolska H., Adamus-Matuszyńska A. Conflicts and Their Solutions in Healthcare Organization. File:///C:/Users/Gumed/AppData/Local/Temp/ZZL(HRM)_2-2011_Saryusz-Wolska_H_Adamus-Matuszynska_A_86-104-1.pdf.

[16] Johansen M.L., Cadmus E. (2016). Conflict management style, supportive work environments and the experience of work stress in emergency nurses. J. Nurs. Manag..

[17] (2020). JBI Manual for Evidence Synthesis. JBI Man. Evid. Synth..

[18] Falcó-Pegueroles A., Lluch-Canut T., Roldan-Merino J., Goberna-Tricas J., Guàrdia-Olmos J. (2014). Ethical conflict in critical care nursing. Nurs. Ethic.

[19] Pishgooie A.H., Barkhordari_Sharifabad M., Atashzadeh-Shoorideh F., Falcó-Pegueroles A. (2018). Ethical conflict among nurses working in the intensive care units. Nurs. Ethic..

[20] Saberi Z., Shahriari M., Yazdannik A.R. (2018). The relationship between ethical conflict and nurses’ personal and organisational characteristics. Nurs. Ethic..

[21] McAndrew N.S., Hardin J. (2020). Giving nurses a voice during ethical conflict in the ICU. Nurs. Ethic..

[22] Bruce C.R., Miller S.M., Zimmerman J.L. (2015). A Qualitative Study Exploring Moral Distress in the ICU Team. Crit. Care Med..

[23] Ramathuba D.U., Ndou H., Ramathuba N.H. (2020). Ethical conflicts experienced by intensive care unit health professionals in a regional hospital, Limpopo province, South Africa. Health SA Gesondheid.

[24] Paprocka-Lipińska A., Drozd-Garbacewicz M., Erenc J., Wujtewicz M., Suchorzewska J., Olejniczak M., Wujtewicz M., Aszkiełowicz H., Dończyk A., Furmanik J. (2019). Potential sources of conflict in intensive care units—a questionnaire study. Anaesthesiol. Intensiv. Ther..

[25] Van Keer R.-L., Deschepper R., Francke A.L., Huyghens L., Bilsen J. (2015). Conflicts between healthcare professionals and families of a multi-ethnic patient population during critical care: An ethnographic study. Crit. Care.

[26] Fernández-Castillo R., González-Caro M., Fernández-García E., Porcel-Gálvez A., Garnacho-Montero J. (2021). Intensive care nurses’ experiences during the COVID -19 pandemic: A qualitative study. Nurs. Crit. Care.

[27] Jia Y., Chen O., Xiao Z., Xiao J., Bian J., Jia H. (2021). Nurses’ ethical challenges caring for people with COVID-19: A qualitative study. Nurs. Ethic..

[28] Gordon J.M., Magbee T., Yoder L.H. (2021). The experiences of critical care nurses caring for patients with COVID-19 during the 2020 pandemic: A qualitative study. Appl. Nurs. Res..

[29] Liu X., Xu Y., Chen Y., Chen C., Wu Q., Xu H., Zhu P., Waidley E. (2021). Ethical dilemmas faced by frontline support nurses fighting COVID-19. Nurs. Ethic..

[30] Donkers M.A., Gilissen V.J.H.S., Candel M.J.J.M., van Dijk N.M., Kling H., Heijnen-Panis R., Pragt E., van der Horst I., Pronk S.A., van Mook W.N.K.A. (2021). Moral distress and ethical climate in intensive care medicine during COVID-19: A nationwide study. BMC Med. Ethic..

[31] Falcó-Pegueroles A., Zuriguel-Pérez E., Via-Clavero G., Bosch-Alcaraz A., Bonetti L. (2021). Ethical conflict during COVID-19 pandemic: The case of Spanish and Italian intensive care units. Int. Nurs. Rev..

[32] Almost J., Wolff A.C., Stewart-Pyne A., McCormick L.G., Strachan D., D’Souza C. (2016). Managing and mitigating conflict in healthcare teams: An integrative review. J. Adv. Nurs..

[33] Schuster R.A., Hong S.Y., Arnold R.M., White D.B. (2014). Investigating Conflict in ICUs—Is the Clinicians’ Perspective Enough?. Crit. Care Med..

[34] Pecanac K., Schwarze M.L. (2018). Conflict in the intensive care unit: Nursing advocacy and surgical agency. Nurs. Ethic..

[35] Fant C. Ethical Dilemmas in Nursing. https://www.nursetogether.com/Career/Career-Article/itemid/2520.aspx.

[36] Shaffer F.A., Curtin L. What Can Employers Do to Increase Nurse Retention?. https://www.myamericannurse.com/wp-content/uploads/2020/08/an8-Turnover-728.pdf.

[37] Bartholomew K. Managing RN/RN and RN/MD Conflict in the ICU: Productive Ways of Communicating. https://healthmanagement.org/c/icu/issuearticle/managing-rn-rn-and-rn-md-conflict-in-the-icu-productive-ways-of-communicating.

[38] Azoulay E., De Waele J., Ferrer R., Staudinger T., Borkowska M., Povoa P., Iliopoulou K., Artigas A., Schaller S.J., Hari M.S. (2020). Symptoms of burnout in intensive care unit specialists facing the COVID-19 outbreak. Ann. Intensiv. Care.

[39] Azoulay E., Pochard F., Reignier J., Argaud L., Bruneel F., Courbon P., Cariou A., Klouche K., Labbé V., Barbier F. (2021). Symptoms of Mental Health Disorders in Critical Care Physicians Facing the Second COVID-19 Wave. Chest.

